# "Saying no is no easy matter" A qualitative study of competing concerns in rationing decisions in general practice

**DOI:** 10.1186/1472-6963-5-70

**Published:** 2005-11-09

**Authors:** Benedicte Carlsen, Ole Frithjof Norheim

**Affiliations:** 1 Health Economics, Bergen, Stein Rokkan Centre for Social Studies, The University of Bergen, Nygårdsgaten 5, 5015 Bergen, Norway; 2 Professor, The Department of Public Health and Primary Health Care, Section for General Practice, The University of Bergen, Kalfarveien 31, 5018 Bergen, Norway

## Abstract

**Background:**

The general practitioner in Norway is expected to ensure equity and effectiveness through fair rationing. At the same time, due to recent reforms of the Norwegian health care sector, both the role of economic incentives and patient autonomy have been strengthened. Studies indicate that modern general practitioners, both in Norway and in other countries are uncomfortable with the gatekeeper role, but there is little knowledge about how general practitioners experience rationing in practice.

**Methods:**

Through focus group interviews with Norwegian general practitioners, we explore physicians' attitudes toward factors of influence on medical decision making and how rationing dilemmas are experienced in everyday practice.

**Results:**

Four major concerns appeared in the group discussions: The obligation to ration health care, professional autonomy, patient autonomy, and competition. A central finding was that the physicians find rationing difficult because saying no in face to face relations often is felt uncomfortable and in conflict with other important objectives for the general practitioner.

**Conclusion:**

It is important to understand the association between using economic incentives in the management of health care, increasing patient autonomy, and the willingness among physicians to contribute to efficient, fair and legitimate resource allocation.

## Background

The demand for health care services is rising in all Western countries and governments are concerned with controlling costs and ensuring a fair allocation of resources [1,2]. The general practitioner (GP) is increasingly regarded as holding a key role in securing equity and effectiveness [3]. In the wake of government concern, a number of studies of rationing and the role of gatekeepers in health care have appeared [4-6].

Rationing can be defined as the allocation of scarce resources between patients with competing needs [7]; it implies the withholding of potentially beneficial health care through deliberate choice or through financial or organisational features of the healthcare system in question [8]. Related to studies of rationing is the concept of *opportunity cost*, which can be defined as *the value of the best alternative which is forgone in order to get or produce more of the commodity under consideration *[9]. In health care decision making this signifies that any use of health care resources should be viewed as denying the opportunity for some other patient to use the money for potentially greater benefit [10].

The view of physicians as rationing agents, commonly accepted by health economists and health administrators, can be contrasted with a more traditional patient advocacy view, where the physician is seen first of all as representing the best interests of each individual patient. Thus, there is often said to be a conflict between the role of physicians as gatekeepers and as patients' advocates. This simple dichotomy is, however, misleading. Many commentators have pointed out that it is possible to hold multiple roles in the physician-patient relationship and that what role one adopts is highly context sensitive [11-13]. In fact, GPs are expected to both take care of the individual patient's need and at the same time take account of common resource use. In Norway, for example, this dual responsibility is clearly stated in the Norwegian Medical Association's ethical guidelines [14].

The gatekeeper role is one of many dilemmas. A Norwegian study from 1993 showed that among a selection of general practitioners, 93% had experienced a conflict between responsibility towards the individual patient and the requirement to manage the health budget [15]. Both British and North American studies of physicians' attitudes to rationing in their practices indicate that GPs are increasingly uncomfortable with the gatekeeper role [5,16,17]. In the case of Norway, we know that Norwegian GPs are reluctant to function as gatekeepers [18], referral rates are increasing [19], and that guidelines for rationing seldom are adhered to [20].

The Norwegian government has expanded the use of economic incentives and market mechanisms in the health sector, while still relying on GPs to filter access to a number of specialised services including both drug and non-drug treatments. In some areas, e.g. the prescription of some common drugs covered by the National Insurance Scheme, the government provides guidelines for rationing. Yet in most instances (e.g. referrals), the GPs are expected to use their best professional judgement to secure an effective and fair allocation of resources.

In 2001 a list-based system was introduced in general practice covering practically the whole population. In addition the payment system was altered from a combination of fee-for-service payment and a practice allowance component, to the current system where the fixed component is replaced by a capitation part. Every GP is granted a fixed remuneration for each person listed, while the rest of the income comes from activity based remunerations and a small out of pocket fee. The Norwegian GPs have no budget responsibility (as e.g. in the UK fund-holding system). The introduction of the patient-list system was seen as a step towards increased patient rights and adequate access to health care. In 1999 The Patients' Rights Act was passed which accentuate the patients' autonomy, e.g. the right to free choice of hospitals and the right to be informed and involved in medical decision making [21]. We have described elsewhere how the new incentive structure enhances competition and motivation for fulfilling patients' expectations and thereby weakening the GPs commitment to the gatekeeper role [22].

There is an ongoing debate among scholars about how to understand GP behaviour in clinical decision making. In principal-agent theory, the GP is seen as a rational actor who maximizes utility, but there has been considerable divergence in which motivators that are considered relevant in the utility function [3,23]. Alternative models criticise the principal-agent theory for ignoring the influence of norms and altruism in physicians' discretionary choices. The bottom line of empiric research seems to be that economic incentives do have an effect on GPs discretionary choices, but other factors, especially medical considerations and professional norms seem to be more influential [23,24]. Some authors have warned that the use of economic incentives in the management of physicians may backfire and ultimately undermine professional norms [25,26].

In order to achieve the goals of efficient and fair resource allocation, it is vital to understand how the agents of rationing, in this case GPs, experience and manage their gatekeeper role. If the gatekeepers find it impossible to act according to common principles for rationing and priority setting, there will be no rationing or rationing will be haphazard and unfair. However, we know little about physicians' experience of and attitude to these reported rationing dilemmas in general practice [16,18].

The aim of this article is to explore how Norwegian GPs experience rationing dilemmas in everyday practice. In the context that our study was conducted, we anticipated economic incentives and patient claims to be of importance in GPs decision making, but we were open for other suggestions from the interview participants and were aiming at finding out what factors where important in the view of the GPs themselves. We were also interested in understanding how one motivating factor is balanced against another.

## Materials and methods

This empirical study relies on data collected in 11 focus group interviews with a total of 81 participants, all of whom were Norwegian primary care physicians. The interviews took place in spring 2002 as part of a national evaluation study in connection with the introduction of a capitation-based patient list system in primary health care.

A purposive sample was recruited from tutorial groups and specialists' continuous education groups in the counties of Hordaland and Oslo. Both rural and urban municipalities are represented (67.5% of the participants had their practice in an urban municipality). The sample was chosen to represent well-known differences and typical practices. In the final sample, 58% were male and length of experience as a GP varied between 0.5 and 27 years with a mean of 11 years. We invited 23 groups to participate by sending a letter by mail to the group co-ordinators, and followed up the invitations with telephone calls. Of these, 13 groups wished to participate, two declined and eight were difficult to reach by telephone. Among the 13 positive answers, 11 groups were interviewed. The groups were homogenous with respect to age and work experience and, with the exception of one group, balanced with respect to gender.

The data were collected through semi-structured focus group interviews and a short questionnaire given to all participants at the start of each interview. The interview guide was tested in the first group interview, after which a number of changes in the wording were decided. After this, following the recommended procedure in the *framework approach *[27], the questions were not changed again. The interviews were conducted by the authors; a social scientist trained in social anthropology and a GP and professor in medical ethics. Interviews lasted between one and two hours, and the discussion was audio taped and transcribed for subsequent analysis. Each interview began with an introduction by one of the researchers who clarified the study's focus and central concepts after which discussion between the participants was encouraged.

The introduction presented our underlying assumption about discretionary choices [28]. The main message was as follows: "Even when practice is based on the methods of evidence-based medicine, numerous grey areas and uncertain indications for treatment remain. Physicians are therefore key actors in rationing decisions and the scope for discretion is wide. It is in these grey zones of discretionary choices that everyday decision making shapes the content and volume of medical practice."

We focused on three types of decisions where there are substantial grey areas and where the scope for discretion is known to be wide:

- The prescription of reimbursed drugs

- The referral of patients to secondary care

- The issuing of sickness certificates

The physicians then discussed how they perceived that different factors influence their professional discretion. We used an interview guide with 12 questions focusing on the informants' view of the gatekeeper role, how to balance the obligation to serve the individual patient vs. rationing and their experience with difficult rationing decisions. We asked specifically of how the relationship with patients and economic incentives affect how they make rationing decisions. As the focus of the study was rationing dilemmas, the participants were encouraged to consider the problematic cases.

### Analysis

The transcripts were coded by hand. Initially the statements in the transcripts of the first four interviews were condensed as keywords and short phrases based on both the original framework and new themes introduced by the informants. This was conducted separately by the two authors. Then the two sets of coded texts were compared and the researchers agreed upon a system of categorisation based on recurrent subjects in the keywords. The analytical steps were inspired both by Kvale's eminent description of coding and categorisation [29] and by Ritchie and Spencer's analytical steps in the framework approach [27].

The quotes presented here are not necessarily the best formulated or the most striking. Rather we have tried to select representative statements that were not too long and that can be understood when separated from the context. We have translated the statements from Norwegian to English.

### Ethical considerations

The project has been reviewed and approved by the Norwegian Social Science Data Service against the privacy and license requirements of the Personal Data Registers Act and the guidelines for research ethics in the social sciences, law and the humanities according to the National Committee for Research Ethics in the Social Sciences and the Humanities. All respondents were informed about anonymity issues, their right to withdraw from the study and the purpose of the study.

## Results

When describing discretionary choices, respondents' answers centred around four major topics:

- The obligation to ration health care

- Professional autonomy

- Patient autonomy

- Competition

In the following section, we report the results of our analysis of the four major issues of special concern. Many of the citations are related to more than one of the major topics. This is quite illustrative of how the different factors seem to be constantly weighed against one another but also intertwined when the informants are describing incidents of making discretionary choices.

### The obligation to ration health care

Participants described many cases of how demanding patients induce them to act as patients' advocate and neglect the gatekeeper role, although some of them stressed that in most instances they have no problems balancing the two roles, for instance through informing and reasoning with the patient. Others stated that they experience a predicament between rationing on society's behalf and offering the best service available to the consulting patient. In the words of one of the participants: *Saying no is no easy matter.*

Below three informants are discussing reasons for giving in to patients' claims for referrals:

Informant 1: *The patients who want a referral to a specialist have made up their mind beforehand that that's what they want. And then I find it quite difficult to explain to them that they don't need it.*

Informant 2: *And it takes twice as much time.*

Informant 3: *I feel in a way that I have to offer good service. I don't want anyone to leave my list. I don't want a bad reputation, do I? I live in the same place that I work and I want to please people.*

(Three participants in a spesialists' group, Oslo)

Many times informants exclaimed that they just can not bring themselves to say no when patients are insistent. Instead of saying no, other strategies are used to evade conflict as this story illustrates:

I often find it hard and very time consuming to get people to change their minds. The most recent example that comes to mind was an elderly lady. I finally ended up saying, "Yes, I think that before we go any further I should refer you to a rheumatologist who can assess your need for this treatment, and then we can talk." It was perhaps a bit cowardly of me. I could have told her, "You won't get that treatment from me and if you want me to be your doctor that's the way it's going to be." But I couldn't face doing that so I referred her; so now she has to go to the rheumatologist and then come back to me and then we'll see. Hopefully he agrees with me.

(Male GP specialist, 26 years of experience as a GP)

Another finding, however, was a lack of awareness among several of the respondents about the need for rationing and the reasons behind government guidelines and regulations. Some said that they do not see the point in rationing when the costs involved are small.

I believe that as GPs we very rarely consider the aspect of public finances, that we for instance refrain from making a referral because of high public costs. We usually use more rational arguments related to what is best for the patient, then we argue that the patient will not benefit much from a certain referral and therefore we will not refer. We try not to think too much that this implicitly means less public expenditure, so that only to a small degree do we make our decisions based on the common good.

(Male GP, 4 years of experience as a GP)

Many were unsure of what are expected of them as gatekeeper. Several seemed to have solved the quandary by concluding that when a GP spends time and effort in trying to convince the patient that there is no indication for a requested intervention, the GP is an active gatekeeper even if the result is that the patient gets his or her own way in the end.

But aren't we gatekeepers when we put up barriers for people by discussing things and putting forward arguments, even when the conclusion is that the person gets what he wants? Isn't that another way of being a gatekeeper?

(Male GP, 3 years experience as a GP)

### Professional autonomy

Most of the informants were concerned about the degree of professional autonomy and freedom in their work, both in rationing decisions and in how to run their practice. Hence the concept of professional autonomy as it is used here is dual, incorporating autonomy in relation to patients and autonomy in relation to the health authorities.

Some stated that the relative freedom of GPs compared to for example hospital physicians was an important reason for their initial career choice. During interviews, some informants initially claimed that their professional autonomy is not at all complicated by patients' demands and government guidelines for rationing, but during the discussions it became evident that many informants are fearful that they are gradually losing their traditional freedom and autonomy in clinical decision making. Many illustrative examples were given of how difficult it is to maintain an autonomous position.

Early in the interviews several informants claimed that, in situations where there is room for discretionary choice, they are influenced neither by patients nor by health authorities.

I would never compromise my own judgement and give patients what they want all the time just to keep them on my list. [...] I have to say that professional concerns always come first, before that sort of selfish need.

(Female GP specialist, 20 years of experience as a GP)

In contrast a few informants explained how they refer to government rules and guidelines because it makes it easier to refuse demands from patients.

If they come in and say that they're depressed and so on, it can be difficult, but it's tidier if you follow the rules. So I prefer to tell them that these are the rules instead of making exceptions in individual cases, because that's a dangerous path.

(Female GP specialist, 16 years of experience as a GP)

There were several informants who conveyed a feeling of conflict between professional autonomy and rules and guidelines set by central health authorities. One example much discussed was the use of statins and anti-hypertensives:

Question: *Do you find that the limits the government has set are clear when it comes to anti-hypertensives and statins?*

Informant: *No. I tend to stick to my own, let's say, professional judgement and not to any official limitations. That is definitely the case.*

(Female GP specialist, 15 years of experience as a GP)

*Another matter is the kind of public finance decision about which level of spending to choose. How much money does Norway as a society want to spend on anti-hypertensives, and how much does Russia want to spend? It's a political decision. The reason why it's sensible to have a spending limit in a country is of course that if all physicians have different limits and some of them are treating patients too aggressively while others are not treating aggressively enough, then the cost-benefit of the use of medicines is less, and it becomes more or less random which group of patients gets help. If there had been an absolute limit, and the limit was set at a reasonable level, maybe not a cholesterol level of 8.2 but let's say 7.5, then the physicians would have to respect the rule and the patients who did not fulfil this criterion but still were eager to get treatment could pay for it themselves. *[The levels as they are set today] *are not respected by GPs, and that may be because the criteria are unrealistic, and we are trying to satisfy our patients.*

(Male GP specialist, 4 years of experience as a GP)

Others said that the rules for remunerated prescribing are perfectly clear and that they never bend them. However, such statements tended to be moderated or even contradicted later in the discussion. Sometimes one of the participants provoked this openness by admitting to bending the rules for remunerated prescriptions or to referring without sufficient medical indications.

A fear of losing professional autonomy because of economic incentives and competition was expressed by many of our informants, although not always in a straightforward or personal manner. Below are two quotes from the same informant, the first early in the interview and the second later as the discussion had accelerated:

1: *Our decisions are always based on our professional judgement. The reform *[the new capitation- and list-based system] *has had no influence whatsoever.*

2: *A relatively serious drawback *[of the reform] *is that you are forced to become a peddler. At the start it made the hairs at the back of my neck stand on end. It feels very unpleasant. Once you've gone along with this premise I think it's a terribly sad situation for our health services as a whole. It has huge consequences ...*

(Male GP, 6 years of experience as a GP)

### Patient autonomy

There were repeated claims that patients have become better informed about their rights as patients, and that they appear increasingly demanding. When discussing why they give in to patients' demands even when the claims are not clearly within what would be defined as medically necessary; many referred to the ideal of patient autonomy and patient centred care which implies respect for patients' subjective experience and sharing decisions with patients.

What we see as trivial and marginal can be very important for the patient for some reason or other that is not immediately clear. When it comes to referrals for instance, it's incredible how manywomen want to see a gynaecologist for no apparent reason. Most are persuaded not to, but some have had an aunt who had ovarian cancer and they tell you that they're worried because of that. One woman told me that she had found a whole new life after she started visiting the gynaecologist once a year. Then it is not marginal after all.

(Female GP, 4 years of experience as a GP)

I have not experienced much conflict when it comes to saying "no", maybe because we manage to rationalise it so that it becomes reasonable to say "yes", i.e. you refer a patient for a CT scan, or something like that, even though it is obvious to you that medically the patient doesn't need it. But they can become quite anxious, and this is one of your regular patients, and you know that one way of getting rid of the problem is to do it even though you know that medically it is absolutely unlikely that they will find anything. And of course there is always a risk that they do find something. I have been asking myself whether I am able to give a categorical "no" to a demanding patient with whom I expect to have a long-term relationship.

(Male GP specialist, 21 years of experience as a GP)

A common explanation for why it is difficult to say no was the need for social approval.

What's quite typical for the way some colleagues work, and maybe sometimes for the way we work too, is that we're interested in getting the patient admitted to the right place, to the right hospitals, to the right specialists, because then we get happy and satisfied patients.

(Male specialist GP, 20 years of experience as a GP)

The participants were also concerned about avoiding conflicts with patients. The physicians described in detail how they manage to avoid conflict by explaining their medical opinion and sharing decisions with patients. The majority claimed that talking with and convincing the patient virtually kept them out of conflict with patients.

Most often, to avoid conflict, I try to get the patient to share my view.

(Male GP, 3 years of experience as a GP)

However, they often give in when the patient is not convinced:

*You might call it gatekeeping if you could explain to him *[a patient] *that he could manage without the CT scan that it ended up with. But it was quite obvious that to him having the scan was a reasonable way of getting rid of the worry. So that is probably the way it works. You could define gatekeeping as pushing it until you see that there is a real need that is reasonable. Then you rarely get into that conflict. But you would get into that conflict if you received a message from the radiology department telling you that the monthly budget was up, but we don't, you know.*

(Male specialist GP, 21 years of experience as a GP)

### Competition

The physicians generally agreed that they find themselves increasingly drawn into a health care market, where patients act as demanding consumers and they as physicians compete to please these consumers.

Some patients put pressure on the physician to write out a sick leave certificate or prescribe when it perhaps isn't necessary. And if you don't do it, you might be pressured and they might leave for another GP.

(Male GP, 2 years of experience as a GP)

For others it was problematic to admit to being influenced by economic incentives. One of the informants initially answered "no" to our question whether the economic and social incentives in the patient list system influenced his discretionary choices, and he continues:

1: *It can't be that one suddenly should treat patients differently in consultations. That would be strange, wouldn't it? Because I believe that would mean that we are driven by organisational moves rather than our own medical understanding.*

(Male GP, 3 years of experience as a GP)

Later in the interview session, the same informant comments on the same question:

2: *I think maybe that GPs are behaving differently *[after new incentives were introduced]. *Why on earth one would do that is of course a question, but ...*

A general impression from the interviews was willingness to departure from adherence to government rules for e.g. reimbursed prescriptions, in order to satisfy the patients:

*I suppose we have all prescribed cortisone creams and asthma medication and things like that as standard prescriptions *[i.e. covered by the National Insurance Scheme]. *Isn't that right? I mean, the combination of regular patients and the demands made by consumers in the health care market, they won't wait or take no for an answer, right? And I am not sure we are good at setting limits to control it.*

(Male specialist GP, 21 years of experience as a GP)

One reason given for not adhering to the remuneration guidelines was that it is pointless to ration if patients can get what they want from somebody else. This argument clearly also touches on the element of competition for patients.

*A new patient came in who had several things she wanted me to look at. I had just given her three prescriptions and then she adds that she just wanted a prescription she had had before, which I didn't know about as she was a completely new patient. So I wrote out a private prescription *[i.e. not covered by the National Insurance Scheme] *for her, and then she says, "But I usually get a standard prescription!" Then her time was up, and I thought "I can't stand listening to all the details and discussing them," so I just said "Fine" and wrote her the standard prescription.*

(Female specialist GP, 15 years of experience as a GP)

### Competing and compatible motivators

A key finding is that the obligation to ration health care is not generally embraced by GPs. Professional norms and respect for patient autonomy on the other hand, are strongly emphasized as integrated and at the very core of their professional judgement. When it comes to the role of economic incentives, the statements are more ambiguous. The idea of economic incentives is sometimes dismissed as an external factor without power to influence GP decision making whatsoever, while some informants state that they are worried that market mechanisms are gradually undermining professional autonomy.

General practitioners in our study find themselves caught in a web of conflicting concerns presented here as the four main topics. Some of these motives are easy to combine and some are often experienced as conflicting. In the paragraphs about rationing and patient autonomy we reported the participants descriptions of how a firm professional autonomy in relation to patients is experienced as a necessary requirement to fulfil the rationing role, because in the current system with universal coverage by a third party payer and free choice of healthcare provider combined with capitation and competition for patients, it is tempting to go along with patients' wishes. At the same time rationing is often presented as in conflict with respect for patient autonomy. On the other hand, as the findings in the paragraph about competition suggest, respect for patient autonomy is easily combined with the situation of competition and the economic incentives of fee-for-service and capitation. So a central line of conflict seems to be how to balance the obligation to ration health care combined with professional autonomy in relation to patients on the one side and the demand for patient autonomy combined with concerns about competition for patients on the other.

## Discussion

Summing up, the central finding was that when the physicians discuss why rationing can be difficult, they emphasize that saying no in face to face relations often is felt uncomfortable and in conflict with other important objectives.

In a health care system with universal coverage and gate keeping through general practice, rationing presupposes a relatively high level of professional autonomy in relation to patients, but this is often in conflict with other ideals and motives faced by the modern physician, such as respect for patient autonomy. This finding supports other studies in Norway and abroad, describing a dilemma between the roles of gatekeeper and the patient's advocate [15]. In addition, there seems to be a lack of understanding of the rationale behind rationing accompanied by a low degree of adherence to government guidelines. There is apparently a lack of clarity in the signals from the health authorities on how principles for priority setting and rationing should be applied in primary care, but part of the explanation seem to lie in the GPs' concern for upholding their professional autonomy in relation to the health authorities.

It is interesting to ask whether physicians' avoidance of explicit rationing is related to the evolving shift in power from GP to patient. According to Daniels [30], already twenty years ago physicians in the US found it difficult to say no to patients. In the NHS/Scandinavian type of health care system patients have been used to GPs acting as gatekeepers, but now the Norwegian system is changing and patients seem to be beginning to demand their rights as consumers. Physicians, both as a group and in their meeting with patients, are experiencing a decline in status and a loss of autonomy. This is a widely recognised phenomenon and a recurring concern in medical professional discourse [31-34]. At a theoretical level the problem is elegantly solved – the modern physician should seek partnership with patients, ground decisions in evidence-based medicine and surrender to transparency and accountability in their practice [2,34-36] – but our results suggest that things are not so simple in practice. When the interviewees discuss how it is sometimes experienced as very difficult to say no to insistent patients, it implies that rationing in practice and especially in face-to-face relationships is quite another task than priority setting and rationing at the macro level. For many GPs, it is problematic even to talk about such divergent and conflicting motives.

Interestingly, there are studies, especially those focusing on patients' experiences that find that patients seldom challenge the physician's autonomy [2]. Some studies of the patient's role claim that patients do not experience themselves as powerful in meetings with the physician [2]. This is also demonstrated in a Norwegian study of the illness experience of Norwegian chronic back pain sufferers [37]. However, there is not necessarily any contradiction in divergent views between physicians and patients. In fact, it has been shown in several studies of doctor-patient communication that whether the physician experiences pressure from the patient is a more powerful predictor of the physician's actions than the actual preferences of patients [38-40]. This might imply, as suggested by Rogers [41], that physicians' stories of demanding patients are sometimes an excuse when the actual explanation is to be found in private economic motives or low awareness of the need for rationing. Elston [36], in her criticism of the deprofessionalisation thesis, also supports this view. She claims that the so-called deprofessionalisation and alleged loss of power of the medial profession is exaggerated. It is not so much a question of whether physicians are capable of carrying out rationing as whether they want to. Likewise Frankel and colleagues [42] suggest that part of the claims of unsatisfied demand in the NHS spring from professional self interest of the NHS-employees.

The ethics of the profession still emphasise that to be a physician is not like other jobs, and the altruistic professional is contrasted with the amoral and therefore untrustworthy expert governed by market forces [43,44]. In this context, being a physician is still presented as a vocation to do good, as an art of caring for the individual patient's needs. The physician as rationing agent is less discussed. The introduction of "anti-professional" incentives such as external surveillance and control as well as the incentives of consumerism can be viewed as the start of a negative spiral [36], as some of the interviewees also indicate. At the same time the modern physician is expected to respect patients' or consumers' autonomy in clinical decision making and to practise patient-centred medicine, which is associated with more satisfied patients and a better health outcome [44,45]. According to this influential research, in clinical decision making the physician should also consider the patient's experience of the symptoms and include such subjective factors in addition to the biomedical facts. Keeping this context in mind it is not difficult to comprehend the informants' ambiguous attitudes toward increasing consumerism in health care and why professional autonomy might be experienced as a competing concern to patient autonomy.

### Strengths and limitations of the study

Focus groups were chosen in preference to other qualitative methodologies because they have been shown to be a useful method of revealing attitudes to complex and sensitive topics [46], partly because the more reserved participants are encouraged by participants who are less inhibited [53]. Participants should have a similar background in relation to the topic to be discussed. In a group of peers where the participants are in the majority, it appears that they are more able and willing to clarify and express thoughts that are difficult to reveal in one-to-one interviews [47,48]. This enhances the common ground and clarifies the prevailing opinions within the specific group under study, as well as the rationale behind them. Thus the focus group interview has been reported to have advantages when the purpose is to reveal common norms in groups of peers, as for example professional groups [53]. On the other hand, focus groups may not be suitable for exploring individual opinions that diverge from group norms [53]. The group context will sometimes scare participants from voicing disagreement.

The main purpose of this study was to reveal common experiences and professional culture regarding the gatekeeper role rather than to investigate individual cases or register the whole spectre of opinions. We therefore decided that focus groups would be more appropriate than for instance individual interviews. we did, however, decide to register how much each participant spoke during the focus group meetings to get an indication of the level of dissent voiced in the groups. We found that the proportion of speech varied extensively between participants, and that those who expressed the strongest opinions spoke the most. This picture is in agreement with our general impressions of the interviews, and it appears that the participants were not afraid of confronting each other's opinions. While it would also have been an advantage to know what was left unspoken, The GPs seem to have a strong feeling of professional loyalty that might have prevented interviewees from revealing opinions that are at odds with the collegial culture. It is therefore doubtful whether this type of data would have emerged if we had had the opportunity to interview all the participants individually afterwards.

Focus groups have a limited value in drawing conclusions on the distribution of different opinions in the population. The sample of GPs participating in these interviews is similar to the general populations of Norwegian GPs according to a few measurable characteristics, as we have described elsewhere [22]. However this is not a randomised sample and we are not aiming at calculating the proportion of Norwegian GPs that would agree with one or all of the arguments presented here. Rather it is reasonable to expect that our interpretations shed light on some of the important elements that influence the countless daily rationing decisions made by modern GPs.

During the interviews there were several indications that we had struck on a sensitive subject with our focus on discretionary choices and how physicians are influenced by patients and economic incentives. In some sense we were questioning their professional autonomy. A fairly recent study by Pearson and Hyams suggests that physicians are reluctant to discuss conflicts of interest in medical decision making [49]. It might be argued that our role in the groups was adding to the complications of a difficult topic. Though not explicitly stated, our introduction and the context of the study, (we were funded by the Ministry of Health through the Norwegian research council to assess how the influence of the patient-list system affects rationing – decisions), gave the participants reason to believe that we could be promoting the need to ration health care and would expect the GPs to be rationing agents in accordance with governmental policy and guidelines.

One indication that the topic was felt to be uncomfortable appears when contrasting the initial answers with the statements made as the participants engaged in group discussion. Several times participants started out by rejecting the focus of the study as irrelevant to their own practice experience. Others said that they did not experience any form of predicament themselves as they were simply practising medicine and were not influenced by economic or other external factors in their discretionary choices. In spite of the slow start of the interviews, we found that attitudes changed throughout each interview from at times quite robust and categorical negative responses to the question of possible dilemmas in medical decision making to a more nuanced and positive response and enthusiastic discussion as the interview proceeded. An equivalent response is reported by Marshall et al. in a focus group study of quality assessment in primary care [50]. Marshall et al. term this "initial vs. considered response". It may well be argued that what we observe is simply a sign of the participants considering the questions in depth for the first time and thus changing their answers. This may also be a characteristic quality of the group process that, together with the comfort of being among peers and outnumbering the researchers, explains why group interviews are an appropriate tool for studying sensitive topics.

We interpret the contradictions and initial unwillingness to discuss discretionary choice as a sign of conflicting ideals, of which the participants are either conscious or not. It is apparent that it is not unproblematic morally to be influenced by economic incentives, and even being influenced by patients' wishes seems to be difficult to acknowledge.

A limitation of the study is that it does not give us any certain knowledge about the actual behaviour of the physicians. Informants may not describe their actions correctly, either because they are unaware of or do not remember their own pattern of actual choices or because they do not wish to reveal them [51] and according to Fernadez et al. [52] GPs consider it legitimate to be influenced by professional standards while to be influenced by financial incentives is not considered legitimate. In this Spanish survey, the responding GPs considered that they were most influenced by the most legitimate factors and least influenced by the least legitimate factors.

A final point to keep in mind is that since rationing as a subject was introduced by the researchers as part of the stated starting point for the group discussions, it is not possible on the basis of these data to estimate how much of the physicians' consciousness is devoted to rationing in their daily practice.

## Conclusion

Based on the discussions within our selection of Norwegian general practitioners, we extract some competing concerns that complicate rationing decisions in primary care and make it difficult to say no to patients who insist on receiving services even when their demands are in conflict with a commitment to legitimate and fair resource allocation. Two central dilemmas are:

• It is often experienced as difficult to make rationing decisions within the context of patient centred medicine

• The current economic incentives do not combine well with making rationing decisions.

Although we do not claim that the informants in this study are representative of GPs everywhere and the findings in this study must be interpreted within the special organisational context of the current Norwegian primary care system, we still believe that it yields interesting knowledge about how different concerns are balanced against one another. The main factors we found influencing these GPs are quite similar to what other studies find both in the Norway and abroad. This and other studies suggest that in a publicly financed health care system we need a better understanding of the relationship between incentives used in the management of primary care, increasing patient autonomy and the willingness among physicians to contribute to efficient, fair and legitimate resource allocation.

## Competing interests

The author(s) declare that they have no competing interests.

## Authors' contributions

BC designed the study. BC and OFN collected and analysed the data. BC drafted the manuscript. BC and OFN revised and finally approved the manuscript.

## Pre-publication history

The pre-publication history for this paper can be accessed here:


